# Supplementary material for: Allosteric regulation of lysosomal enzyme recognition by the cation-independent mannose 6-phosphate receptor
Source: Commun Biol. 2020 Sep 9;3:498. doi: 10.1038/s42003-020-01211-w (PMC7481795; doi:10.1038/s42003-020-01211-w)
Supplement: Supplementary file 1 — Supplemental information [file 42003_2020_1211_MOESM1_ESM.pdf]

Supplementary Figures

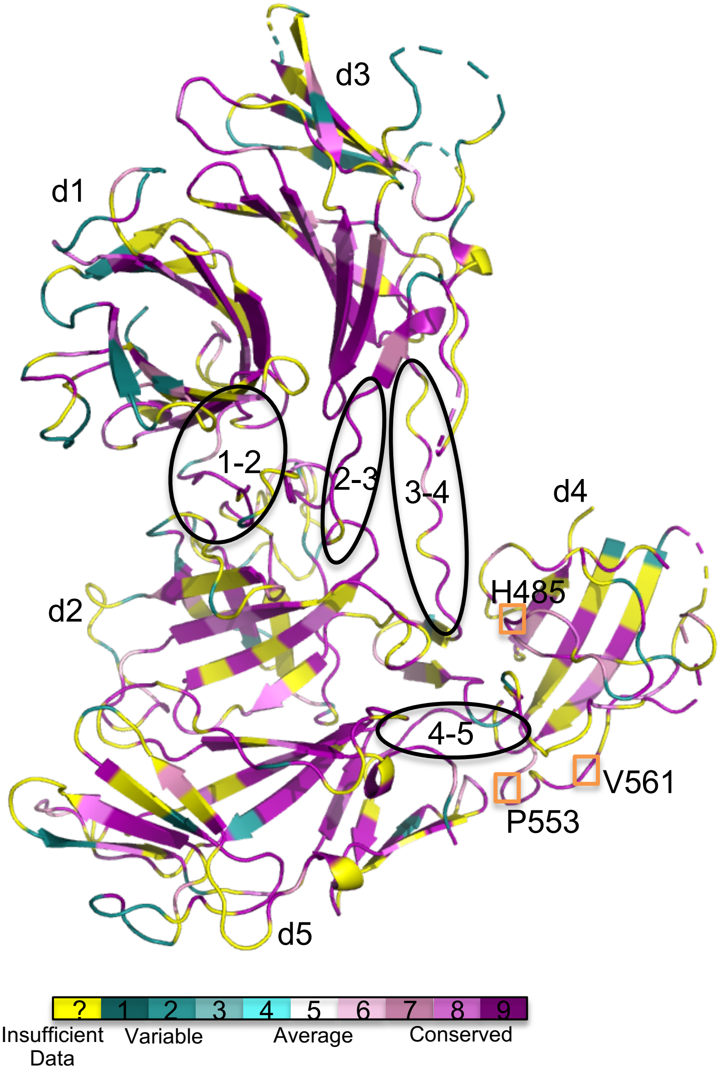

Supplementary Fig. 1. Consurf results showing amino acid species conservation. The PDB 6P8I structure was analyzed via the ConSurf server (https://consurf.tau.ac.il/) to determine regions of amino acid conservation and then mapped onto the model. The individual domains of domains 1-5 are labeled while the linkers between domains are labeled and circled in black. Amino acids are colored by the degree of conservation as defined by ConSurf (conserved, purple; variable, cyan). Residues boxed in peach are highly conserved and discussed in Supplementary Fig. 5.

a

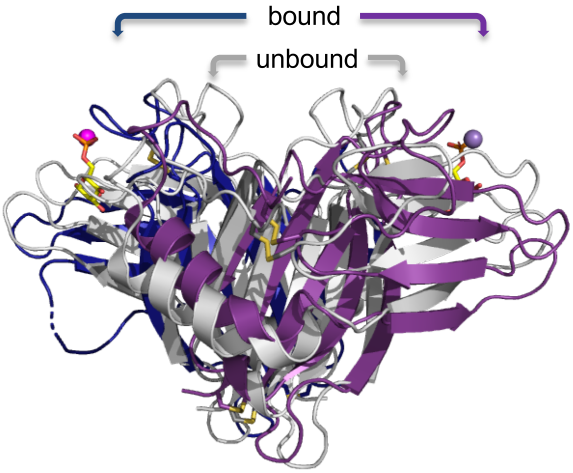

c

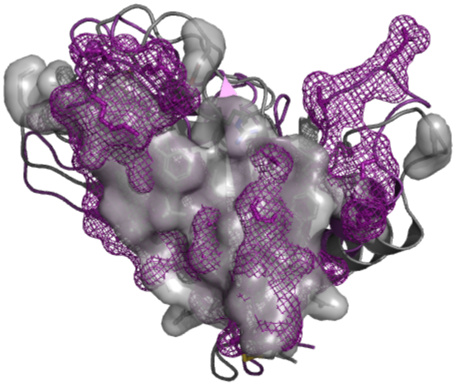

b

Domains	Area (Å2)	CSS*	# H-bonds	# salt bridges
1:3 (unbound d3)<br>(bound d3)	1019<br>370	1.0<br>0.30	10<br>8	2<br>3
2:5	967	0.0	19	10
1:2 (unbound d3)<br>(bound d3)	782<br>860	1.0<br>1.0	8<br>8	3<br>2
2:4 (unbound d3)	503	0.0	4	1
4:5 (unbound d3)	477	0.0	6	6
2:3 (unbound d3)<br>(bound d3)	262<br>717	0.097<br>0.79	8<br>18	4<br>4
CD-MPR (unbound)<br>(bound)	769<br>1044	1.0<br>0.46	11<br>9	0<br>2

*Complex Formation Significance Score

Supplementary Fig. 2. Comparison of CD-MPR and N-terminal 5 domains of CI-MPR. a, Superimposition of Cα atoms of the homodimeric form of CD-MPR in the bound (PDB 2RL8) (monomer 1, dark purple and monomer 2, dark blue) and unbound (PDB 1KEO) (grey) forms. The M6P found in the bound structure is shown in yellow while the manganese ions are shown as pink and purple spheres. **b,** Table of interface areas and CSS values calculated with PISA (https://www.ebi.ac.uk/msd-srv/prot_int/pistart.html). **c,** Comparison of solvent inaccessible interface residues of CD-MPR in the bound (purple mesh) and unbound (grey surface) states highlighting differences in contact areas.

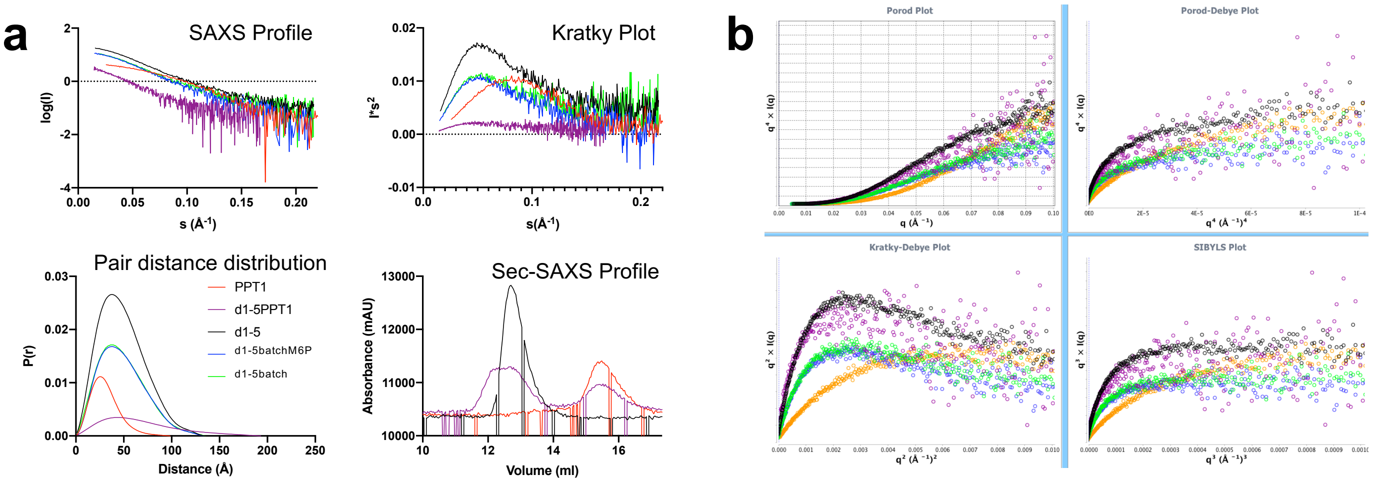

c

	R_g	D_{max}	P_E	Vol	MW	AA MW
	(Å)	(Å)		(Å^3)	kDA	
D1-5 batch	37	101	2.9	140000	87.5	81.3
D1-5 batch w/M6P	37	102	2.9	156000	97.5	81.3
D1-5	38	115	2.9	145000	90.6	81.3
PPT1	23	59	1.3	59900	37.4	31.3
D1-5+PPT1	41	114	2.8	203000	126.9	112.6

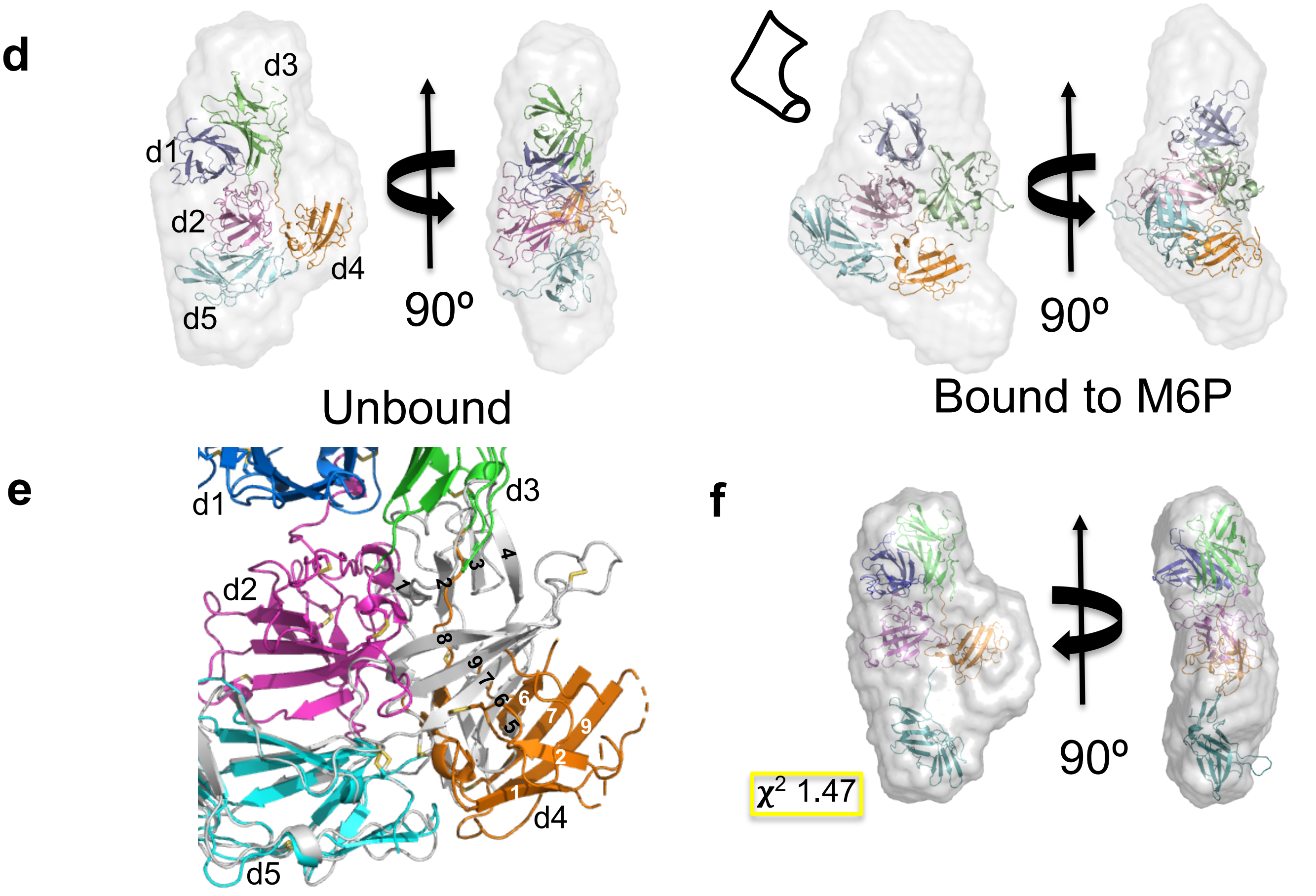

Supplementary Fig. 3. SAXS data analysis. a, Plots of the SAXS Profile, Kratky Plot, Pairwise distance distribution (SEC-SAXS: domains 1-5 black, PPT1 orange, and complex of CI-MPR domains 1-5 with PPT1 purple; batch mode samples(domains 1-5 green, domains 1-5 with M6P, blue). The elution profile of domains 1-5 with PPT1 from a G200-Increase column (domains 1-5 black, PPT1 orange, and complex of CI-MPR domains 1-5 with PPT1 purple). **b,** Flexibility plots colored as in panel (a). **c,** Table of SAXS derived values: R_g, radius of gyration; D_{max}, maximum dimension; P_E, Porod exponent; Vol, envelope volume: MW, molecular weight calculated from envelope volume; AA MW, molecular weight calculated from masses of amino acids of protein sequence. **d,** PDB 6P8I crystal structure modeled into SAXS derived envelope for domains 1-5 in the absence of M6P (unbound). Domains 1-5 with modified domain 3 structure inserted into the envelope derived from data collected in the presence of M6P (bound to M6P). **e,** Superimposition of Cα atoms of domain 5 (cyan) of human domains 1-5 (PDB 6P8I) onto corresponding atoms of endogenous bovine CI-MPR purified from liver (PDB 6UM2) (grey) illustrating the relative position of domain 4 in each structure (PDB 6P8I, orange; PDB 6UM2, grey). Strands of domain 4 are labeled in each case to aid in visualization the 180° domain rotation this domain undergoes. **f**, MultiFoXS calculated model where the domain 4-5 linker is allowed to be flexible, resulting in an ~60Å movement of domain 5 relative to domain 2 in (d). Corresponding χ^2 value for the curve is shown.

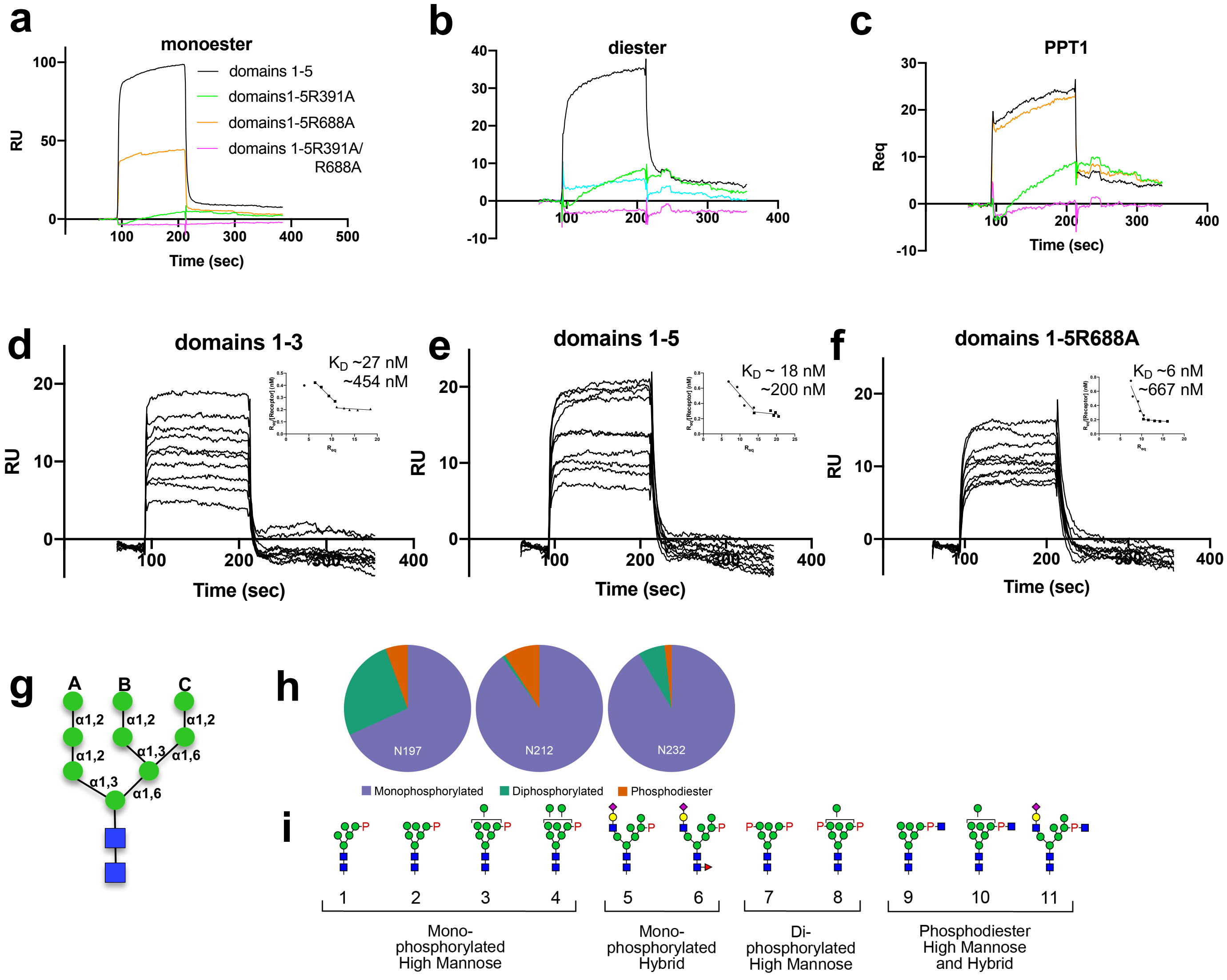

Supplementary Fig. 4. Characterization of glycans of recombinant PPT1 by SPR and mass spectrometry. SPR sensorgrams for either domains 1-5 truncated protein or CRD binding mutants (domains 1-5R391A, domains 1-5R688A, domains1-5R391A/R688A; replacement of residue essential for phosphomannosyl binding in domain 3 (R391) and domain 5 (R688) results in loss of carbohydrate binding) (120 nM) flowing over GAA-phosphomonoester **(a)** or GAA-phosphodiester **(b)** and PPT1 **(c).** Sensorgrams for domains 1-3 (**d**), domains 1-5 (**e**) or domains 1-5R688A (**f**) protein (10 to 120 nM) flowing over PPT1 surface. Scatchard Plot insets with two binding events and associated K_D (-1/slope) values indicated. **g,** Schematic illustration of a typical high mannose glycan with linkages and arms are labeled. Green circles represent mannose residues, while blue squares represent N-acetylglucosamine residues. **h,** Pie charts depicting percentages of glycans found in recombinant PPT1 protein. **i**, Schematic diagram representing various forms of glycans identified for each of the glycosylation sites of PPT1 by mass spectrometry.

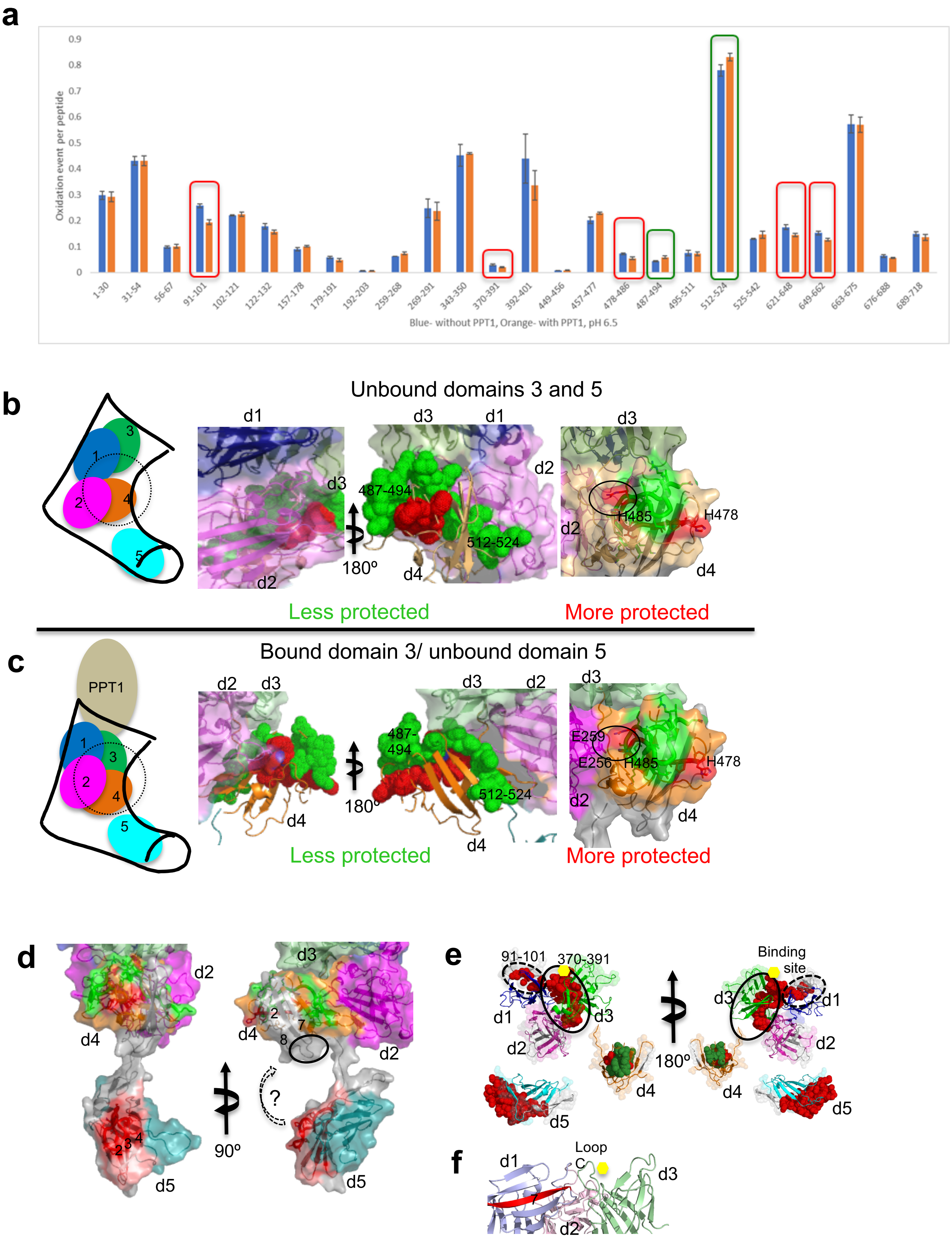
a
Oxidation event per peptide
0.9
0.8
0.7
0.6
0.5
0.4
0.3
0.2
0.1
0
1-30
31-54
56-67
91-101
102-121
122-132
157-178
179-191
192-203
259-268
269-291
343-350
370-391
392-401
449-456
457-477
478-486
487-494
495-511
512-524
525-542
621-648
649-662
663-675
676-688
689-718
Blue- without PPT1, Orange- with PPT1, pH 6.5
b
Unbound domains 3 and 5
d1
d3
d2
487-494
512-524
180º
d4
H485
H478
Less protected
More protected
c
Bound domain 3/ unbound domain 5
PPT1
487-494
512-524
E259
E256
H485
H478
Less protected
More protected
d
d5
90º
?
e
91-101
370-391
Binding site
180º
f
Loop C

Supplementary Fig. 5. The FPOP results of (a) tryptic peptide fragments identified in the absence (blue) and presence (orange) of PPT1) were mapped onto our SAXS models. a, Peptides undergoing statistically significant ($p \leq 0.05$) changes between the two conditions are boxed (red, more protected in presence PPT1; green, less protected in the presence of PPT1). FPOP data mapped onto our unbound model of domains 1-5 (**b**) is compared to the same data mapped onto our SAXS derived model representing a bound domain 3 and an unbound domain 5 (**c-d**). Peptides 487-494 and 512-524 transition on ligand binding from a protected interface with domain 3 in the unbound position (**b**) to a more solvent-exposed position in the bound structure (**c, less protected, rotated**). Only N-terminal residues H478, F479, and F480 and C-terminal residues H485 and R486 are solvent-exposed in the unbound model with the three N-terminal residues remaining solvent-exposed in the bound model **(b, more protected, and c, more protected)**. **c, more protected**, Species conserved H485 becomes part of the interface with domain 2 forming a hydrogen bond (black circle) with mainly conserved E256 and E259, and it most probably accounts for the change in reactivity of this peptide. **d**, Domain 5 has two peptides that show protection from oxidation in the presence of ligand compared to the absence (peptides 621-648 (strands 2 and 3) and 649 to 662 (strand 4). Grey surfaces represent regions where no FPOP data is available. **d, rotated**, shows a possible region of interaction between protected domain 5 peptides and region of domain 4 (black circle) with high species conservation but no FPOP data available. **e,** The protected peptide 370-391 (domain 3) (circled in black) is located in the binding site of domain 3 (yellow hexagon) and encompasses the previously identified highly conserved, essential residue for ligand binding residue R391. Another protected peptide 91-101 is labeled (dashed black circle). **f,** Peptide 91-101 is located on strand 7 on the C-terminal back β-sheet of domain 1, and when domain 3 is in the bound position, this strand is across from the ligand-binding site (yellow hexagon).

a

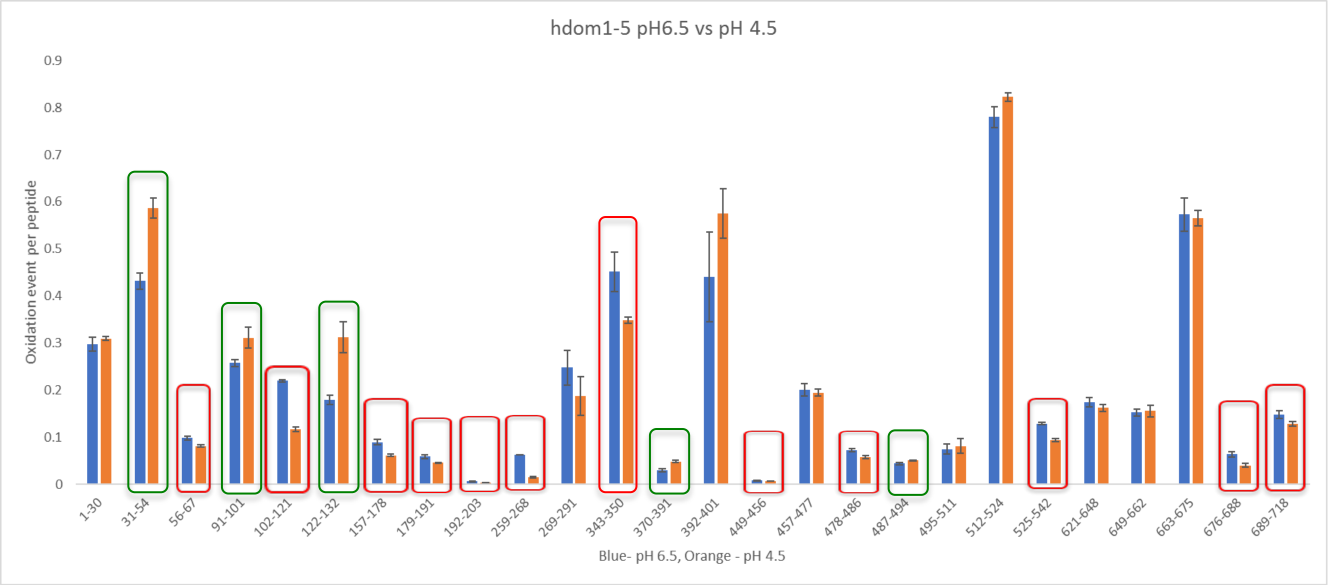

b

Domains	Area (Å²) (%∆)	CSS*	# H-bonds	# salt bridges
1:3 (unbound d5)<br>(low pH)	1019<br>1094 (107)	1.0<br>0.0	10<br>11	2<br>2
2:5 (unbound d5)<br>(low pH)	967<br>1142 (118)	0.0<br>0.0	19<br>13	10<br>9
1:2 (unbound d5)<br>(low pH)	782<br>852 (109)	1.0<br>0.0	8<br>10	3<br>2
2:4 (unbound d5)<br>(low pH)	503<br>847 (168)	0.0<br>0.0	4<br>4	1<br>5
4:5 (unbound d5)<br>(low pH)	477<br>300 (63)	0.0<br>0.0	6<br>3	6<br>1
2:3 (unbound d5)<br>(low pH)	262<br>218 (83)	0.097<br>0.0	8<br>6	4<br>3
3:4 (unbound d5)<br>(low pH)	112<br>425 (380)	0.0<br>0.0	0<br>7	1<br>7

*Complex Formation Significance Score

Supplementary Fig. 6. The FPOP results (a) tryptic peptide fragments identified at pH 6.5 (blue) and pH 4.5 (orange). Peptides undergoing statistically significant ($p \leq 0.05$) changes between the two conditions are boxed (red, more protected at pH 4.5; green, less protected at pH 4.5). **b,** Table of interface areas and CSS values calculated with PISA (https://www.ebi.ac.uk/msd-srv/prot_int/pistart.html).

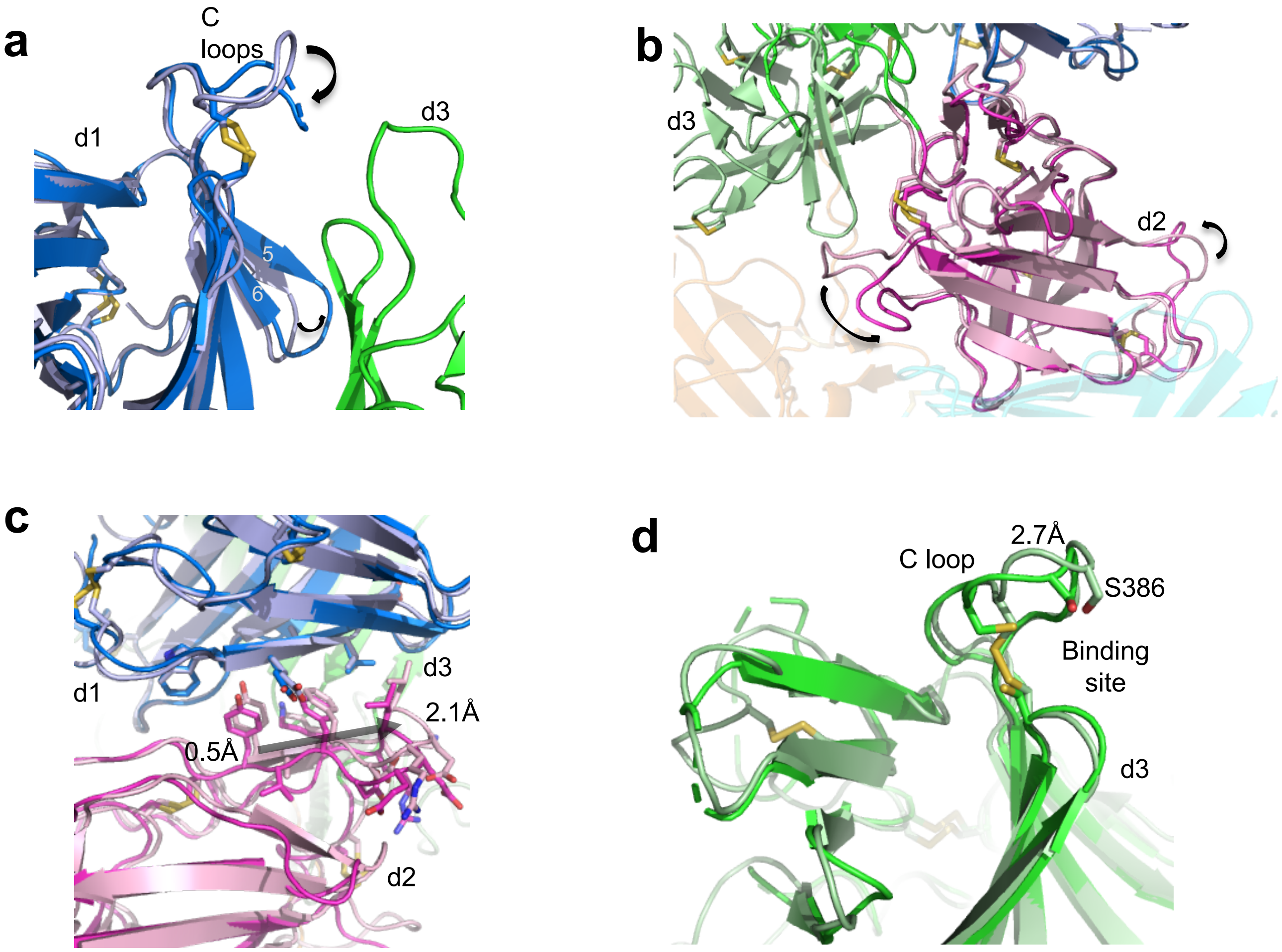

Supplementary Fig. 7. Loop movements in the presence of ligand (PDB 1SYO) could relay message of binding to remote regions: possible allosteric mechanism. **a**, Loop C (between strands 6 and 7) of domain 1 relocates 6.4 Å towards loop C of domain 3 in the presence of ligand (PDB 1SYO) (dark blue) compared to its position in the absence of ligand (PDB 6P8I)(light blue). The loop between strands 5 and 6 of domain 1 moves towards domain 3 by 4.2 Å in the presence of ligand (dark blue), allowing it to form contacts with residues in domain 3 (Supplementary Fig. 2b). **b**, Domain 2 has multiple loops experiencing conformational changes as well as modest changes in the β-strands of the N-terminal ('front') sheet relative to the strands of the C-terminal ('back') sheet in the presence (PDB 1SYO)(magenta) and absence of ligand (PDB 6P8I) (light pink). **c**, The position of domain 2 changes relative to domain 1 by 0.5 to 2.1 Å along the grey arrow (at the junction point between domains 1, 2, and 3) in the presence (1SYO) versus absence of ligand (PDB 6P8I). **d**, The Cα of S386, located at the tip of loop C and essential for ligand binding, moves away from the binding cavity by 2.7 Å in the unbound structure (PDB 6P8I) (light green) compared to the bound structure (PDB 1SYO)(dark green). Position of disulfide bonds are indicated (yellow) in all panels.

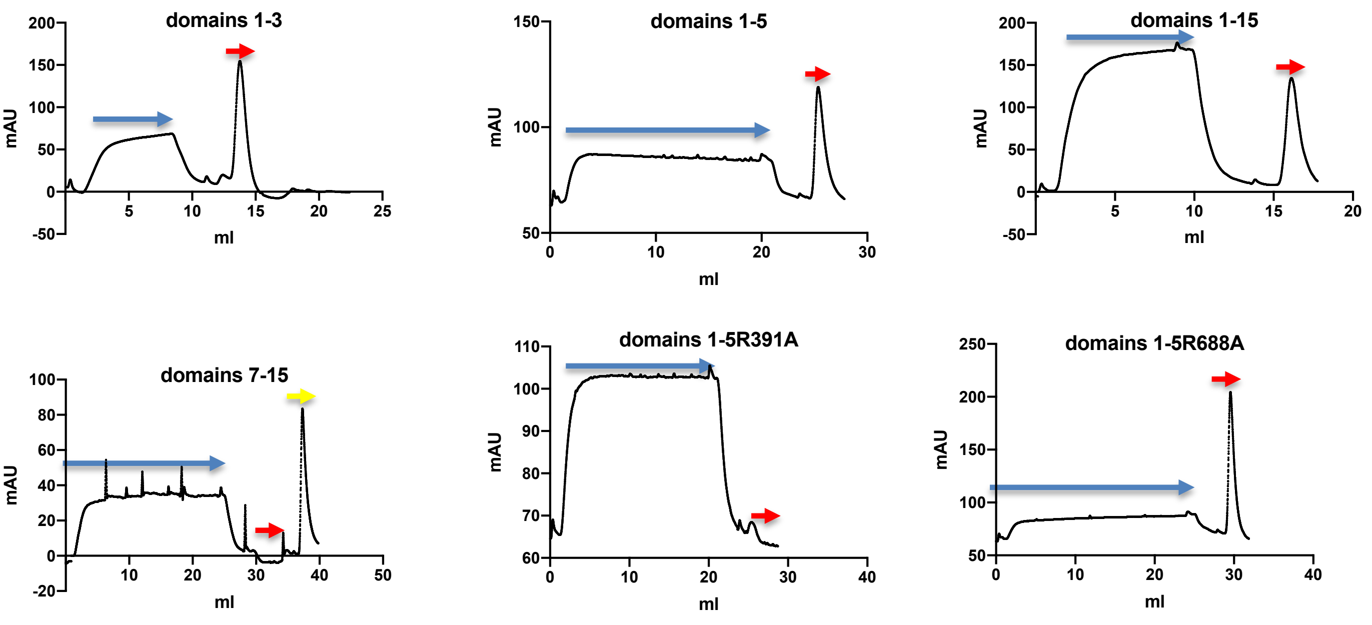

Supplementary Fig. 8. Chromatograms of CI-MPR constructs on the PPT1 affinity column monitored by Absorbance$_{280}$. Blue arrows indicate the loading of the CI-MPR sample onto 1 ml PPT1 affinity column generated by primary amine coupling. Red arrow indicates elution of the column with pH 4.5 buffer, and a yellow arrow indicates elution of the column with buffer containing 10 mM M6P. Replacement of the residue essential for phosphomannosyl binding in domain 3 (R391) and domain 5 (R688) results in loss of carbohydrate binding by the respective domain in the domains 1-5 construct.
