# Supplementary material for: Allosteric regulation of lysosomal enzyme recognition by the cation-independent mannose 6-phosphate receptor
Source: Commun Biol. 2020 Sep 9;3:498. doi: 10.1038/s42003-020-01211-w (PMC7481795; doi:10.1038/s42003-020-01211-w)
Supplement: Supplementary file 2 — Reporting Summary [file 42003_2020_1211_MOESM2_ESM.pdf]

nature research

Corresponding author(s): Linda J. Olson and Nancy M. Dahms

Last updated by author(s): Jul 27, 2020

Reporting Summary

Nature Research wishes to improve the reproducibility of the work that we publish. This form provides structure for consistency and transparency in reporting. For further information on Nature Research policies, see our Editorial Policies and the Editorial Policy Checklist.

Statistics

For all statistical analyses, confirm that the following items are present in the figure legend, table legend, main text, or Methods section.

n/a	Confirmed	
☐	☒	The exact sample size (n) for each experimental group/condition, given as a discrete number and unit of measurement
☒	☐	A statement on whether measurements were taken from distinct samples or whether the same sample was measured repeatedly
☐	☒	The statistical test(s) used AND whether they are one- or two-sided<br>*Only common tests should be described solely by name; describe more complex techniques in the Methods section.*
☒	☐	A description of all covariates tested
☒	☐	A description of any assumptions or corrections, such as tests of normality and adjustment for multiple comparisons
☒	☐	A full description of the statistical parameters including central tendency (e.g. means) or other basic estimates (e.g. regression coefficient) AND variation (e.g. standard deviation) or associated estimates of uncertainty (e.g. confidence intervals)
☒	☐	For null hypothesis testing, the test statistic (e.g. F, t, r) with confidence intervals, effect sizes, degrees of freedom and P value noted<br>*Give P values as exact values whenever suitable.*
☒	☐	For Bayesian analysis, information on the choice of priors and Markov chain Monte Carlo settings
☒	☐	For hierarchical and complex designs, identification of the appropriate level for tests and full reporting of outcomes
☒	☐	Estimates of effect sizes (e.g. Cohen's d, Pearson's r), indicating how they were calculated

Our web collection on statistics for biologists contains articles on many of the points above.

Software and code

Policy information about availability of computer code

Data collection	Xtalview was used to collect crystallographic data. SPR data was collected using BiaControl software
Data analysis	The following software was utilized during these studies: HKL2000, ATSAS, Phenix, CCP4i, pymol, prism, Coot and Chimera

For manuscripts utilizing custom algorithms or software that are central to the research but not yet described in published literature, software must be made available to editors and reviewers. We strongly encourage code deposition in a community repository (e.g. GitHub). See the Nature Research guidelines for submitting code & software for further information.

Data

Policy information about availability of data

All manuscripts must include a data availability statement. This statement should provide the following information, where applicable:

- Accession codes, unique identifiers, or web links for publicly available datasets
- A list of figures that have associated raw data
- A description of any restrictions on data availability

All data generated or analyzed during this study are included in the published article and its Supplementary Information. The X-ray crystal structures and structure factors of human domains 1-5 of CI-MPR at pH 5.5 and 7.0 have been deposited in Protein Data Bank under accession codes PDB 6P8I and PDB 6V02. SAXS data have been deposited at SASDBD with codes: SASDHL4 (N-terminal domains 1-5 of the cation-independent mannose-6-phosphate receptor (CI-MPR)), SASDHM4 (N-terminal domains 1-5 of the cation-independent mannose-6-phosphate receptor (CI-MPR) from SEC-SAXS), SASDHN4 (N-terminal 5 domains of the cation-independent mannose-6-phosphate receptor (CI-MPR) bound to mannose 6-phosphate (M6P)), SASDHP4 (Palmitoyl-protein thioesterase 1 (PPT1)), and SASDQ4 (N-terminal domains 1-5 of the cation-independent mannose-6-phosphate receptor (CI-MPR) in complex with palmitoyl-protein thioesterase 1 (PPT1)).

Field-specific reporting

Please select the one below that is the best fit for your research. If you are not sure, read the appropriate sections before making your selection.

☒ Life sciences ☐ Behavioural & social sciences ☐ Ecological, evolutionary & environmental sciences

For a reference copy of the document with all sections, see nature.com/documents/nr-reporting-summary-flat.pdf

Life sciences study design

All studies must disclose on these points even when the disclosure is negative.

Sample size	Sample sizes were not predetermined by statistical methods
Data exclusions	No data was excluded
Replication	All attempts at replication succeeded
Randomization	Ramdomization was not utilized or required
Blinding	Blinding was not required

Reporting for specific materials, systems and methods

We require information from authors about some types of materials, experimental systems and methods used in many studies. Here, indicate whether each material, system or method listed is relevant to your study. If you are not sure if a list item applies to your research, read the appropriate section before selecting a response.

Materials & experimental systems

n/a	Involved in the study	
☒	☐	Antibodies
☐	☒	Eukaryotic cell lines
☒	☐	Palaeontology and archaeology
☒	☐	Animals and other organisms
☒	☐	Human research participants
☒	☐	Clinical data
☒	☐	Dual use research of concern

Methods

n/a	Involved in the study	
☒	☐	ChIP-seq
☒	☐	Flow cytometry
☒	☐	MRI-based neuroimaging

Eukaryotic cell lines

Policy information about cell lines

Cell line source(s)	Sf9 cells were purchased from Expression Systems and PPT1 overexpressing CHO cells were obtained from UT-Southwestern
Authentication	cell lines were authenticated by supplier and no official authentication was done.
Mycoplasma contamination	Cell lines were tested by supplier and have not been further tested.
Commonly misidentified lines (See ICLAC register)	none
